# C-Reactive Protein as a Prognostic Factor for Human Osteosarcoma: A Meta-Analysis and Literature Review

**DOI:** 10.1371/journal.pone.0094632

**Published:** 2014-05-06

**Authors:** Jian-Hua Yi, Dong Wang, Zhi-Yong Li, Jun Hu, Xiao-Feng Niu, Xiao-Lin Liu

**Affiliations:** The Upper Limb Orthopedic Department of Huang Pu Award, The First Affiliated Hospital of Sun Yat-Sen University Guangzhou, China; Aligarh Muslim University, India

## Abstract

**Background:**

Osteosarcoma is the most common primary bone cancer in growing adolescents and young adults. The prognostic role of C-reactive protein (CRP) in patients with osteosarcoma is not fully investigated. The purpose of this study is to perform a meta-analysis and literature review on the role of CRP in osteosarcoma and to assess the potential role of serum CRP as a prognostic factor for patients with osteosarcoma.

**Methods:**

A detailed literature search was made in Medline for related research publications written in English. Methodological quality of the studies was also evaluated. The data were extracted and assessed by two reviewers independently. Analysis of pooled data were performed, risk ratio (RR) and corresponding confidence intervals (CIs) were calculated and summarized respectively.

**Results:**

Final analysis of 397 patients from 2 eligible studies was performed. Combined RR of CRP expression suggested that the raised serum CRP level had an adverse prognostic effect on overall survival of patients with osteosarcoma (n = 397 in 2 studies; RR = 0.35; 95% CI: 0.18–0.68; p = 0.002). In the uni- and multivariate survival analysis, response rate and CRP levels were the only independent prognostic variables.

**Conclusions:**

The results of this meta-analysis suggest that CRP expression confers a worse prognosis in patients with osteosarcoma. Large prospective studies are necessary to provide solid data to confirm the prognostic significance of CRP.

## Introduction

The two most common primary bone malignancies, osteosarcoma (OS) and Ewing sarcoma (ES), are both aggressive, highly metastatic cancers that occur in children and young adults [1], [2]. OS originates from primitive bone-forming mesenchymal cells and ranks the eighth most common cancer of childhood in the United States. About 15–20% patients present with overt lung metastases at initial diagnosis and 40% patients develop metastases at a later stage [3], [4]. Despite multidisciplinary treatments including surgery, neoadjuvant and adjuvant chemotherapy, the overall five-year survival rate for OS remains 60–70% [5]. The mechanism underlying OS metastasis is still limited, neovascularization, invasion, anoikis resistance, chemoresistance, and evasion of the immune response are considered to be required for progression and metastasis in OS [6]. OS metastasis causes a pejorative prognosis [7], [8], [9]. In order to improve the clinical outcomes for patients with poor prognosis, it is urgent to find new diagnostic and therapeutic approaches to identify high-risk patients and block metastasis in this disease.

C-reactive protein (CRP) is an acute phase protein, which is produced principally by hepatocytes with serum level correlated with systemic inflammation [10]. It belongs to a highly conserved phylogenetically ancient family called pentraxins, which have five identical non-covalently linked subunits [11]. CRPs are characterized by their homopentameric structure and calcium-dependent ligand-binding affinity for the phosphocholine (PhC) moiety [12], [13]. The wide distribution of PhC in polysaccharides of pathogens and in cellular membranes enables CRP to recognize a range of pathogenic targets as well as membranes of damaged and necrotic host cells [13]. Expression of CRP is principally induced at the transcriptional level by interleukin-6 (IL-6), which can be synergistically enhanced by IL-1 and tumor necrosis factor (TNF). In humans, the CRP level is low (0.1–0.5 *µ*g/ml) under normal conditions, but can increase its serum level up to 100-fold during systemic inflammation [12], which probably allows CRP to be the single most useful molecule for monitoring acute-phase reactions. CRP is a nonspecific but sensitive marker of inflammation. Increase CRP level is considered to be an important risk factor for inflammatory disease [14], atherosclerosis, myocardial infarction, peripheral vascular disease, and ischemic stroke [15], [16], [17], [18], [19], [20]. It is positively associated with weight loss, anorexia-cachexia syndrome, extent of disease, and recurrence in advanced/high-grade cancer [21], [22]. Its role as a predictor of survival has been shown in multiple myeloma, melanoma, lymphoma, ovarian, renal, pancreatic, and gastrointestinal tumors [17], [21], [23], [24], [25].

In this study, we sought to perform a meta-analysis and systematic review to evaluate whether CRP was correlated with outcome of OS patients. A pool of research data published between 2007 and 2013 on the study of the correlation between CRP and patients' survival, histological response to adjuvant chemotherapy and recurrence in OS were analyzed. Our analysis indicated that CRP had an unfavorable impact on OS patients' overall survival.

## Methods

### Search strategy and selection criteria

Medline, Pubmed and Web of Science were searched using the search terms: ‘c-reactive protein’, ‘prognosis’ and ‘osteosarcoma’. All searched data were retrieved. Authors' bibliographies and references of selected studies were also searched for other relevant studies. The most complete study was chosen to avoid duplication if same patient populations were reported in several publications.

Two reviewers estimated the eligibility of studies independently by reviewing titles and abstracts found by the search. The inclusion criteria included: 1) CRP evaluated in the primary OS tissues; 2) relationship displayed between CRP serum levels and OS clinicopathological variables or prognosis; 3) CRP serum levels detested by latex-enhanced immuno-turbidimetric test; 4) publications written in English; 5) data provided sufficient information to analyze risk ratio (RR) and 95% confidence interval (CI). The exclusion criteria included: 1) duplicate data set on the same patient populations; 2) articles published in non-English; 3) case reports, editorials, letters, reviews and conference abstracts. The detailed information of 13 relevant citations was listed in Table 1.

**Table 1:** All 13 citations identified through database searching.

Author	Included/excluded	Comments
Mulrooney DA et al. 2013 [98]	Excluded	Pilot study of vascular health in survivors of osteosarcoma
Nakamura T et al. 2013 [5]	Included	Determine whether circulating CRP predicts survival in bone sarcoma
Peng TI et al. 2012 [99]	Excluded	Osteosarcoma 143B cells were utilized for study of mitochondrial dynamics
Fujiwara T et al. 2011 [100]	Excluded	A study of macrophage infiltration in prediction of human ewing sarcoma
Funovics PT et al. 2011 [22]	Included	A study of CRP as independent prognostic factor for survival in osteosarcoma patients
Jansson AF et al.2009 [101]	Excluded	A study of clinical score for nonbacterial osteitis in patients
Schwinger W et al. 2005 [102]	Excluded	A study of effect of interleukin-2 treatment in pretreated solid tumor patients including osteosarcoma
Patino WD et al. 2005 [103]	Excluded	A study of biomarkers such as osteosarcoma (FOS) gene in atherosclerosis
Das T et al. 2003 [97]	Excluded	A study of glycosylation in human CRP under different pathological conditions
Trapani S et al. 2000 [104]	Excluded	A study of incidence of occult cancer in children presenting with musculoskeletal symptoms
Kleinerman ES et al. 1995 [105]	Excluded	A study of combined therapy on patients with relapsed osteosarcoma
Asano T et al. 1993 [106]	Excluded	A study of a novel biologic agent for osteosarcoma therapy
Kleinerman ES et al. 1992 [107]	Excluded	Phase II study of liposomal muramyl tripeptide in osteosarcoma

### Data extraction and study assessment

Two reviewers (JY, DW) extracted data from selected studies independently. Any discontent was discussed and reached a consensus for all issues. The following items were collected from each study: first author's name, year of publication, country, number of patients, age, sex and CRP expression. Duplicated studies were avoided by checking the authors' name of the institutions.

### Statistic analysis

Hazard ratio (HR) and its variance for each individual study were extracted or analyzed based on the published data according to the methods described previously [26], [27]. A HR>1 was regarded as a risk factor for worse survival in patients with elevated CRP serum levels. This impact of CRP on survival was regarded as statistically significant if the corresponding 95% CI for the summary HR did not overlap 1 unit. Odds ratios (OR) were utilized to measure the relationship of CRP and histological response to neoadjuvant chemotherapy, serum CRP level and tumor size. All P values were two sided. Statistical analyses were conducted using GraphPad Prism (GraphPad Software Inc., LaJolla, California; version 4.00, 2003).

## Results

### Study selection and characteristics

**Figure 1:**
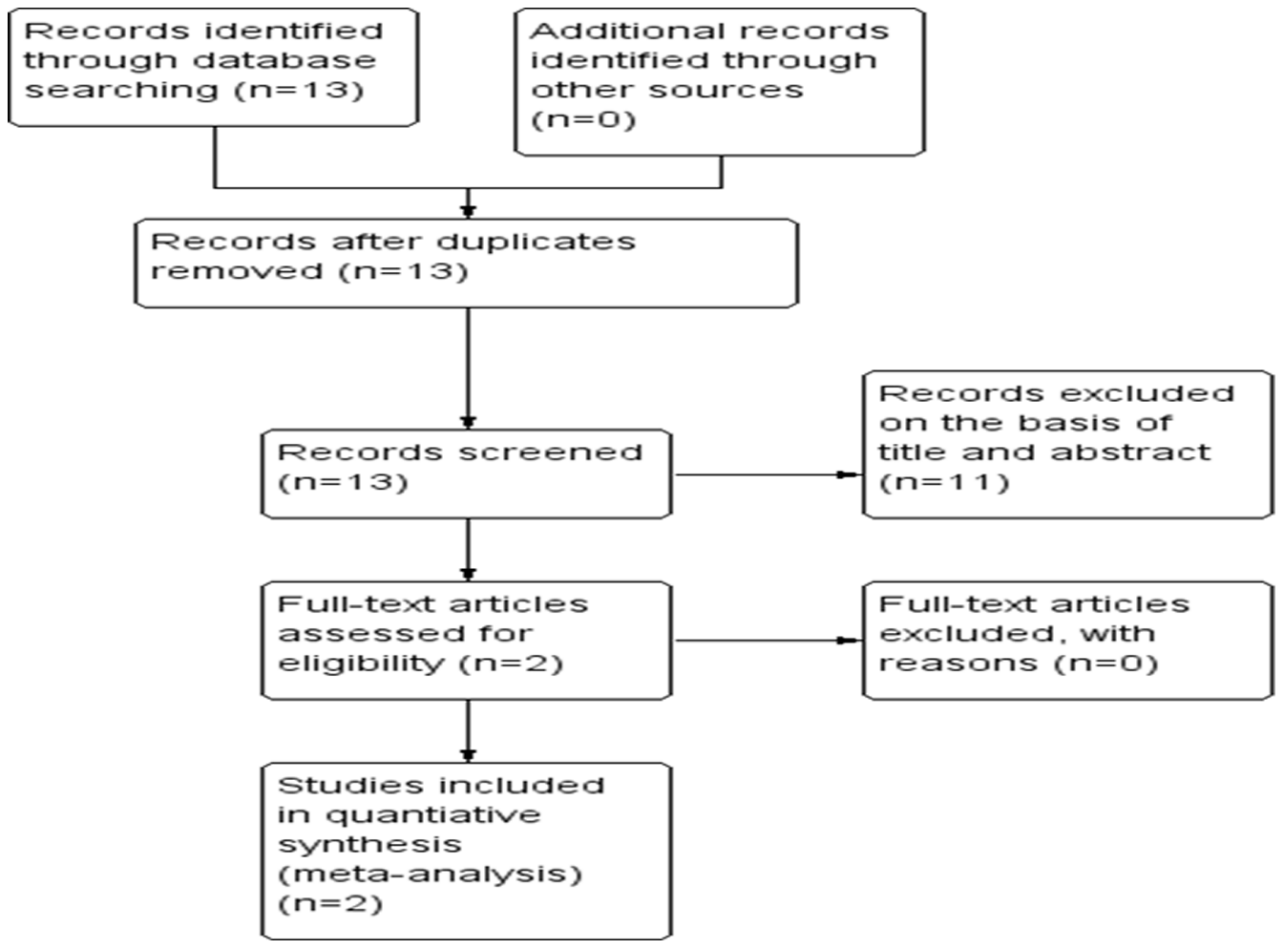
Flow chart of study selection.

13 relevant citations were selected for initial review utilizing search strategies as described above. Of these, 11 were initially excluded after reading the abstracts (Figure 1). As a result, the systematic literature search generated a total of 2 datasets [5], [22] and 397 patients for ultimate analysis. These data were published from 2011 to 2013 to fit the inclusion criteria for our meta-analysis.

A HR on 5 year disease-specific survival could be extracted from these studies, respectively. The survival data were summarized from 2 eligible studies [5], [22] and analyzed the correlation between CRP and histological response to neoadjuvant chemotherapy.

### Methodological quality of the studies

2 selected studies [5], [22] were evaluated by two reviewers with high levels of methodological quality (>6 stars) according to Newcastle-Ottawa quality assessment scale [28].

### Impact of CRP on EFS of OS patients

**Figure 2:**
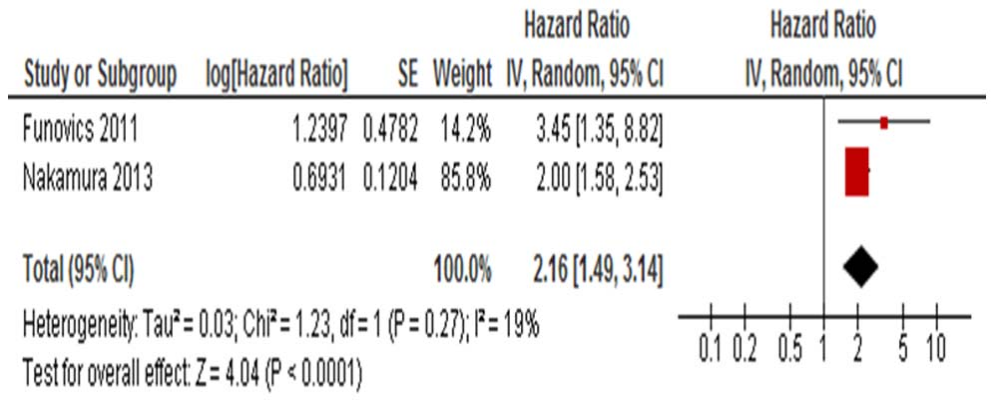
Forest plot showing the association between CRP level and disease-specific survival of osteosarcoma. The summary HR and 95% CIs were shown (according to the random-effects estimations).

**Figure 3:**
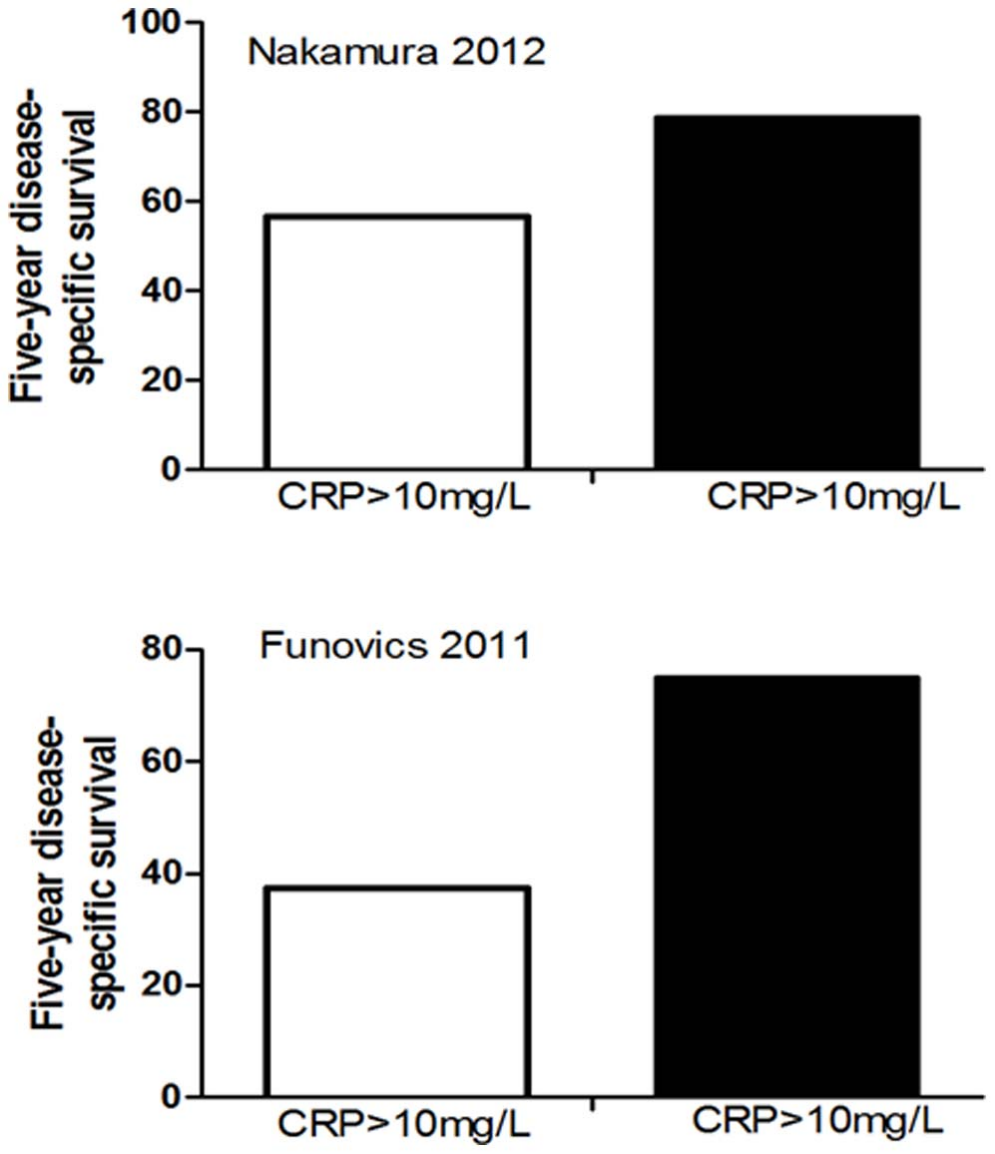
Representative figure of the correlation between survival and CRP levels.

Two studies [5], [22] with a total of 397 OS patients regarding CRP and disease-specific survival were meta-analyzed. Because of heterogeneity (*I* ^2^ = 19%), a random effect model was used in this analysis. The pooled HR was 2.16, illustrating that CRP was significantly correlated with the poor survival of OS patients (Figure 2). The representative figure of the correlation between survival and CRP levels were shown as Figure 3.

### CRP and histological response to neoadjuvant chemotherapy

Two of the selected studies [5], [22] consisting of 397 patients dealt with the correlation between CRP and histological response to neoadjuvant chemotherapy. Due to heterogeneity (*I* ^2^ = 19%), a random effect model was selected for analysis. In the uni- and multivariate survival analysis, response rate and CRP levels were the only independent prognostic variables (Table 2).

**Table 2:** Univariate and multivariate analysis for predictive factors for survival (CI, confidence interval).

Variable	Univariate p		Multivariate p		Hazard risk(95% CI)	
	Funovics	Nakamura	Funovics	Nakamura	Funovics	Nakamura
Sex	0.281	0.92	0.410		1.5 (0.6–3.7)	
Age	0.003	0.6	0.003		1.1 (1.0–1.1)	
Tumor size	0.153	0.002	0.267	0.1	1.0 (1.0–1.1)	0.598 (0.32 to 1.115)
Histological subtype	0.052		0.519		0.6 (0.1–3.3)	
Tumor grade		0.002				
Response to chemotherapy	0.002	0.04	0.024		10.8 (1.4–85.6)	
C-reactive protein	<0.001	<0.0001	0.031	0.02	1.4 (1.0–1.8)	0.598 (0.333 to 0.892)

### Assessment of publication bias

Due to the less number of studies selected in our meta-analysis, we did not draft funnel plot to display publication bias.

## Discussion

CRP is a representative acute phase reactant due to its rapid production and short half-life in circulation. CRP is mainly produced by hepatocytes in the liver in response to inflammatory cytokines, the level of CRP increases when there is inflammation throughout the body. Extrahepatic production of CRP has also been found in tumor cells, monocytes, lymphocytes and neuron as a part of local inflammation, but the amount is too little to affect serum CRP [21]. CRP is a sensitive but non-specific serum biomarker induced by infectious and non-infectious processes [29] including cancer and tissue damage [21]. In addition to its role as a very sensitive indicator of current disease activity for inflammation, the role of serum CRP has recently been re-evaluated by extending its clinical use to the diagnosis of cardiovascular diseases as well as risk prediction of cancer [21].

The linkage of inflammation and cancer has been extensively investigated since the first study reported by Virchow et al [30]. Actually inflammation is the seventh hallmark for cancer initiation and progression, it represents the linkage between intrinsic factors (oncogenes, genome instability) and extrinsic factors (immune and stromal components) [31]. Cancer cells in tumor tissues are always embedded by a persistent inflammatory state, called tumor microenvironment. The leucocytes, lymphocytes and macrophages along with cytokines/chemokines in tumor microenvironment serve as mediators to induce genomic instability, epigenetic changes and subsequent malignancy phenotype of cancer cells [32], [33]. CRP and other acute phase proteins are induced to be synthesized in hepatocytes by cytokines/chemokines and released into circulation. The circulating CRP acts on tumor cells, which lead to tumor cell lysis and facilitate tumor progression [21].

In most prevalent studies, serum CRP levels have been demonstrated to be highly elevated in cancer patients compared to healthy individuals. The prognostic significance of serum CRP was found in various cancer patients and in the advanced stage of cancers, including renal cell carcinoma [34], [35], [36], [37], [38], [39], [40], bladder [41], [42], [43], [44], breast [45], [46], [47], [48], [49], [50], stomach [51], , colon and rectum [56], , head and neck [62], [63], [64], esophagus [25], [65], [66], [67], [68], [69], prostate [70], [71], [72], [73], [74], lung [75], [76], [77], [78], [79] and pancreatic cancer [80], [81]. The high CRP levels have also been associated with aggressive diseases and poor survival outcomes for these malignancies. Its role as a predictor of survival has been shown in multiple myeloma, melanoma, lymphoma, ovarian, renal, pancreatic, and gastrointestinal tumors [23]. Its predictive ability for the survival and individualized therapy of patients with advanced cancers can be improved by combining CRP with other parameters such as tumor necrosis factor (TNF), serum albumin, and serum B12 [21], [82]

With similarity of the role of CRP in other malignancies, elevated level of CRP is also associated with poor prognosis of soft-tissue sarcoma patients [5], [83], [84]. This current review and meta-analysis demonstrated that CRP had an unfavourable impact on disease-specific survival of OS patients. Funovics et al [22] have revealed that pre-operative circulating CRP levels before tumor resection are associated with disease-specific outcomes, that is, patients with CRP levels higher than the cut-off value (1 mg/dl) had a significant worse survival. Therefore, the level of circulating pre-operative CRP was considered to be an independent prognostic factor for survival in patients with high-grade OS [22]. Similar observations were obtained for pre-treatment CRP by Nakamu et al, the elevated levels of pre-treatment CRP are demonstrated to be correlated with a decreased disease-specific survival and an increased rate of local recurrence in patients with a sarcoma of bone [5]. Although pre-treatment serum CRP is a significant prognostic factor and a monitor of tumor aggressiveness in OS patients, it does not apply to Ewing's sarcoma and chondrosarcom.

The reasons why CRP levels correlated with prognosis in tumor patients including OS remain to be determined. An experimental data obtained using transgenic mouse models provides a possible explanation, which showed that serum CRP levels are involved in host defenses against infections [85], [86], [87]. The strong correlation between CRP levels and cancer risk and/or poor prognosis may be due to (1) causality: elevated CRP levels cause cancer, (2) reverse causality: occult cancer increases CRP levels, (3) or confounding: a third factor, e.g. inflammation, increases both CRP levels and the risk of cancer [17], [88]. In addition, the chronic elevation of CRP observed in obese subjects may potentiate leptin resistance, contributing to the carcinogenesis, tumor progression and poor outcomes of tumor patients [89], [90], [91].

The CRP gene is located on the chromosome 1(1q21–q23). The generation of CRP in hepatocytes is induced at the transcriptional level by IL-6, and can be synergistically increased by IL-1β. The synergistic induction of CRP gene expression by IL-6 plus IL-1 requires transcriptional factors CCAAT/enhancer binging protein (C/EBP family α/β/δ) [92], [93], as well as signal transducers and activators of transcription (STAT3). The nuclear factor κB (NF-κB) subunits p50 and p65 through protein kinase C signaling pathway also participates in cytokine-induced CRP synthesis [94], [95]. In addition, transcriptional complex formation of c-Fos, STAT3, and hepatocyte NF-1 alpha is essential for cytokine-driven CRP gene expression [96]. As a result, the interaction of these transcriptional factors induce maximal production of CRP in hepatocytes. Alternatively the unique glycosylated molecular variants of CRP can be induced and subsequently influence it biological function in different clinical pathological conditions [97].

It's still unclear that the relationship between CRP and cancer is a causal one, since so many conditions can elevate CRP levels without increasing cancer risk. Some studies have been conducted to determine if elevated CRP levels and cancer are directly related. Many clinical findings reveal that CRP tends to increase in cancer patients, but the direct relation has not yet been established. The increase in CRP levels could be due to cancerous illness causing inflammation in the body or vise versa. Irrespective of relatively small number of patients, as well as the fact that the analysis was retrospective, the current meta-analysis indicated that the CRP levels can be used as a key indicator of prognosis for osteosarcoma. Nevertheless, additional research in the future especially larger prospective studies will be needed to provide more definitive answers to confirm these results. Overall, measurement of plasma CRP is simple, rapid, cost effective, suitable and provides valuable information for patients with cancer. We believe that development of agents decreasing CRP levels will provide a promising benefit in the prevention and treatment of many different types of human malignancies.

## Supporting Information

Checklist S1 PRISMA checklist.
(DOC)
